# Supplementary material for: Mental Health among Higher Education Students during the COVID-19 Pandemic: A Cross-Sectional Survey from Lithuania
Source: Int J Environ Res Public Health. 2021 Dec 2;18(23):12737. doi: 10.3390/ijerph182312737 (PMC8657419; doi:10.3390/ijerph182312737)
Supplement: Supplementary file 1 [file ijerph-18-12737-s001.zip › ijerph-1435919-supplementary.pdf]

Supplementary data

Questionnaire

1. What is your age? ____

2. What is your sex?
   - o Male
   - o Female

3. What is your study field?
   - o Social sciences (e.g., law, political science, psychology, economics, management, sports)
   - o Biomedical sciences (e.g., biology, medicine, odontology, pharmacy)
   - o Physical sciences (e.g., mathematics, physics, biochemistry, geology, informatics, geography)
   - o Technological sciences (e.g., logistics, chemistry, computer engineering)
   - o Arts (e.g., music, art, theater, architecture, audiovisual art)
   - o Humanities (e.g., philosophy, philology, history, art history)

4. During your studies you live:
   - o Alone
   - o With your family
   - o With other students or peers
   - o With your partner

5. Do you agree with the following statements?

	Strongly disagree	Disagree	Neither agree, nor disagree	Agree	Strongly agree
During COVID-19 pandemic my physical health got worse	O	O	O	O	O
During COVID-19 pandemic I feel more anxiety than usually	O	O	O	O	O
During COVID-19 pandemic I feel more sadness than usually	O	O	O	O	O
During COVID-19 pandemic my academic	O	O	O	O	O

performance got worse					
During COVID-19 pandemic I have no comfortable place to study	O	O	O	O	O
During COVID-19 pandemic I find it more difficult to focus on my studies	O	O	O	O	O
During COVID-19 pandemic my personal relationships got worse	O	O	O	O	O
During COVID-19 pandemic my income became lower	O	O	O	O	O
During COVID-19 pandemic my career prospects got worse	O	O	O	O	O

6. Were you infected with COVID-19?
- o Yes
- o No

7. Were you hospitalized because of COVID-19?
- o Yes
- o No

8. Was any of your family members infected with COVID-19?
- o Yes
- o No

9. Was any of your family members hospitalized because of COVID-19?
- o Yes
- o No

10. Do you usually see a solution to problems and difficulties that other people find hopeless?
- o Yes, often
- o Yes, sometimes
- o No

11. Do you usually feel that your daily life is a source of personal satisfaction?

- Yes, often
- Yes, sometimes
- No

12. Do you usually feel that the things that happen to you in your daily life are hard to understand?
- Yes, often
- Yes, sometimes
- No

After reading each statement, answer how you were feeling over the past week.

13. I feel tense or 'wound up'
- Most of the time
- A lot of the time
- From time to time, occasionally
- Not at all

14. I still enjoy the things I used to enjoy
- Definitely as much
- Not quite so much
- Only a little
- Hardly at all

15. I get a sort of frightened feeling as if something awful is about to happen
- Very definitely and quite badly
- Yes, but not too badly
- A little, but it doesn't worry me
- Not at all

16. I can laugh and see funny side of the things
- As much as I always could
- Not quite so much now
- Definitely not so much now
- Not at all

17. Worrying thoughts go through my mind
- A great deal of the time
- A lot of the time
- From time to time, but not too often
- Only occasionally

18. I feel cheerful
- Not at all
- Not often
- Sometimes
- Most of the time

19. I can sit at ease and feel relaxed
- Definitely
- Usually
- Not often
- Not at all

20. I feel as if I am slowed down
- Nearly all the time
- Very often
- Sometimes
- Not at all

21. I get sort of frightened feeling like 'butterflies' in the stomach
- Not at all
- Occasionally
- Quite often

- Very often

22. I have lost interest in my appearance
- Definitely
- I don’t’ take as much care as I should
- I may not take quite as much care
- I take just as much care as ever

23. I feel restless as I have to be on the move
- Very much indeed
- Quite a lot
- Not very much
- Not at all

24. I look forward with enjoyment to things
- As much as I ever did
- Rather as I ever did
- Definitely less than I used to
- Hardly at all

25. I get sudden feeling of panic
- Very often indeed
- Quite often
- Not very often
- Not at all

26. I can enjoy a good book or radio or TV program
- Often
- Sometimes
- Not often
- Very seldom

27. How do you evaluate your health?
- 5 Very good
- 4 Good
- 3 Average
- 2 Poor
- 1 Very poor

Please answer the questions related to the risk of suicide.

28. Have you ever thought of suicide?
- Yes, I had thoughts of committing suicide
- Yes, I have engaged in creating suicidal plans
- Yes, I tried to commit suicide
- No (if you chose this answer, please end the survey)

29. During the last 12 months, have you experienced sadness from day to day for at least 2 weeks and did not want to do anything?
- Yes
- No

30. During the last 12 months, have you thought of committing suicide?
- Yes
- No

31. During the last 12 months, have you engaged in creating suicidal plans?
- Yes
- No

32. During the last 12 months, have you tried to commit suicide?
- Yes
- No
